# Nitrogen deposition accelerates soil carbon sequestration in tropical forests

**DOI:** 10.1073/pnas.2020790118

**Published:** 2021-04-12

**Authors:** Xiankai Lu, Peter M. Vitousek, Qinggong Mao, Frank S. Gilliam, Yiqi Luo, Benjamin L. Turner, Guoyi Zhou, Jiangming Mo

**Affiliations:** ^a^Key Laboratory of Vegetation Restoration and Management of Degraded Ecosystems, South China Botanical Garden, Chinese Academy of Sciences, Guangzhou 510650, China;; ^b^Center of Plant Ecology, Core Botanical Gardens, Chinese Academy of Sciences, Guangzhou 510650, China;; ^c^Department of Biology, Stanford University, Stanford, CA 94305;; ^d^Department of Biology, University of West Florida, Pensacola, FL 32514;; ^e^Center for Ecosystem Science and Society, Northern Arizona University, Flagstaff, AZ 86011;; ^f^Smithsonian Tropical Research Institute, Apartado 0843-03092 Balboa, Ancon, Republic of Panama

**Keywords:** below-ground carbon sequestration, soil carbon storage, atmospheric nitrogen deposition, nitrogen biogeochemistry, global changes

## Abstract

Terrestrial ecosystem carbon (C) sequestration plays an important role in ameliorating global climate change. While tropical forests exert a disproportionately large influence on global C cycling, there remains an open question on changes in below-ground soil C stocks with global increases in nitrogen (N) deposition, because N supply often does not constrain the growth of tropical forests. We quantified soil C sequestration through more than a decade of continuous N addition experiment in an N-rich primary tropical forest. Results showed that long-term N additions increased soil C stocks by 7 to 21%, mainly arising from decreased C output fluxes and physical protection mechanisms without changes in the chemical composition of organic matter. A meta-analysis further verified that soil C sequestration induced by excess N inputs is a general phenomenon in tropical forests. Notably, soil N sequestration can keep pace with soil C, based on consistent C/N ratios under N additions. These findings provide empirical evidence that below-ground C sequestration can be stimulated in mature tropical forests under excess N deposition, which has important implications for predicting future terrestrial sinks for both elevated anthropogenic CO_2_ and N deposition. We further developed a conceptual model hypothesis depicting how soil C sequestration happens under chronic N deposition in N-limited and N-rich ecosystems, suggesting a direction to incorporate N deposition and N cycling into terrestrial C cycle models to improve the predictability on C sink strength as enhanced N deposition spreads from temperate into tropical systems.

## Significance

Forest soil carbon (C) storage plays a central role in sequestrating atmospheric CO_2_ on timescales from centuries to millennia. However, our current understanding of soil C sequestration in response to N deposition mainly focuses on mid-to-high latitudes in the Northern Hemisphere, where N supply typically constrains forest growth. We lack data about changes in soil C stocks in tropical forests, where most ecosystems are N-rich or N-saturated. Using more than a decade of continuous N addition experiment and a meta-analysis, we found that excess N deposition can significantly increase soil C in N-rich tropical forests. However, enhanced C sequestration in tropical soils is not a good reason to justify excess N emissions to the atmosphere.

With the globalization of anthropologically elevated nitrogen (N) deposition (1–3), ecosystem C sequestration can be stimulated in many places, because N limitation of net primary productivity (NPP) is widespread (4, 5). However, N is relatively abundant in many tropical forests, and experiments demonstrate that N supply does not limit NPP in such N-rich forests (1, 4). Moreover, previous studies on forest C sequestration overwhelmingly emphasized plant productivity rather than soil stocks as sink for C (6–11). Soil is the largest pool of terrestrial organic C in the biosphere, and more than half of soil C is stored in forest ecosystems (12). Accordingly, C sequestration as soil organic matter could be quantitatively more important than vegetation for forest C budgets (8).

Our current understanding of soil C sequestration in response to N deposition in forests has several limitations. First, unlike biomass C sequestration, responses of forest soil C sequestration to N deposition remain inconclusive. Many studies on soil C dynamics indicate that N deposition can increase soil C sequestration by reducing the decomposition of plant litter and soil organic matter (13–15), inhibiting soil respiration (16), or changing microbial enzymatic activity (14, 17). Conversely, other studies reported that long-term N application did not affect soil C sequestration (18), whereas Van Miegroet and Jandl (19) suggested that N addition can deplete soil C pool through microbial respiration linked to transformation of excess N. The contradictory evidence points to the necessity of further examination of how soil C sinks respond to increased N deposition (3, 20). Second, most studies have been conducted in mid-to-high latitudes in the Northern Hemisphere (16, 18, 20, 21), where most forest ecosystems are N-limited, and an increase in N supply can enhance NPP and aboveground litter production (4, 9, 11, 21, 22). Until now, however, we have lacked data about changes in soil C stocks with increased N supply in tropical forests, where ecosystems are more often N-rich (1, 4). This lack of information has led to a debatable assertion of ecosystem C neutrality with N deposition in many tropical forests (21, 23–25). In fact, we do not know what N will do to C storage in N-rich ecosystems because there is a large class of such systems for which there is little information.

Here, we experimentally tested the influence of elevated N deposition on soil C sequestration in an N-rich tropical forest, using more than a decade of N addition to experimental plots established in a lowland primary forest at the Dinghushan Biosphere Reserve (DHSBR) in southern China, which has received high rates of ambient N deposition (e.g., >30 kg N⋅ha^−1^⋅y^−1^ in precipitation) for several decades (26). We first quantified the contribution of long-term N addition to below-ground soil C stocks. To further clarify the mechanisms on which soil C storage was changed, we evaluated soil density fractions, because these fractions are closely related to soil C stability and its potential for long-term preservation. The protection of organic matter in soils generally increases with the increasing density of SOM fractions, or with increasing association with mineral particles in the heavy fraction of soils (27). To characterize the biochemical composition of organic matter residing in mineral soil fractions, we employed solid-state ^13^C-NMR spectroscopy to determine the relative abundance of alkyl C, O-alkyl C, aromatic C, and carboxyl C. Moreover, to identify whether there is a general pattern of N-induced soil C sequestration in the tropics, we further combined this field evidence with a meta-analysis of N addition experiments to quantify the responses of soil C storage in tropical forests to N additions.

## Results and Discussion

### Soil C Sequestration after a Decade of N Addition at an N-Saturated Tropical Forest.

**Fig. 1.**
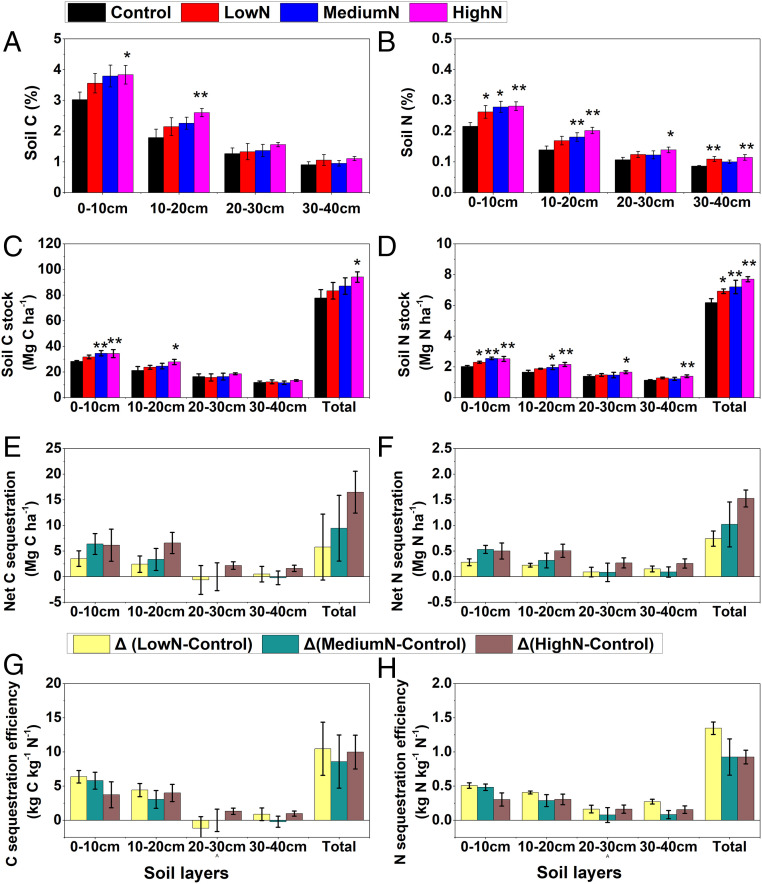
Effects of long-term N addition on soil C and N at 0- to 40-cm soil layers in the N-saturated tropical forest of South China. (*A*) Soil C concentrations. (*B*) Soil N concentrations. (*C*) Soil C stocks. (*D*) Soil N stocks. (*E*) Net C sequestration. (*F*) Net N sequestration. (*G*) C sequestration efficiency (C sequestration rate per unit of nitrogen addition). (*H*) N sequestration efficiency (N sequestration rate per unit of nitrogen addition). Note: Soils were sampled after 11 y of N addition; the single (*) and double asterisks (**) indicate that there are significant differences between N treatments and the controls at *P* < 0.1 and *P* < 0.05 levels, respectively; net soil C and N sequestration generally show increasing trends with greater rates of N addition, while soil C and N sequestration efficiency show decreasing trends; values are means with SE.

Soil C and N concentrations were comparable between treatments at the beginning of N addition (*SI Appendix*, Fig. S1). However, 11 y of N addition (50 to 150 kg N⋅ha^−1^⋅y^−1^) greatly increased soil C and N concentrations in the primary tropical forest at the Dinghushan reserve (Fig. 1 *A* and *B* and *SI Appendix*, Fig. S1). Nitrogen addition significantly increased soil C and N sequestration by 7 to 21% and 12 to 25%, respectively, over the controls for the whole soil layers in this forest (Fig. 1 *C* and *D*), whereas soil bulk density remained comparable among treatments (*SI Appendix*, Fig. S2*A*). Net soil C and N sequestration increased with greater rates of N addition, mainly in the upper 20 cm (Fig. 1 *E* and *F*), where soil C and N sequestration efficiency (C or N sequestration rate per unit of nitrogen addition) decreased with increased N addition (Fig. 1 *G* and *H*). For the whole soil profile, soil C sequestration efficiency ranged from 8.6 to 10.5 kg C⋅kg^−1^⋅N. In temperate forests, a review of published studies showed that soil C sequestration efficiency varied widely, ranging from 3 to 25 kg C⋅kg^−1^ N added and representing 10 to 100% of total C accumulation observed in trees and soils (13, 21, 22), in close agreement with our findings. Using stoichiometric scaling, however, de Vries et al. (10) estimated soil C sequestration efficiency as 5.4 kg C⋅kg^−1^ N for tropical forests, lower than our findings.

In the temperate forests that have received most of the study to date, the sink for soil C sequestration is typically coupled with plant productivity and aboveground litter inputs. We found that, however, long-term N addition affected neither tree growth and litterfall production (26), nor the standing mass of forest floor layer (*SI Appendix*, Table S1). Because the net soil C budget is determined by the differences between C inputs as source and outputs and inputs did not differ, we conclude that decreases in C outputs are mainly responsible for the observed patterns, and identify two potential mechanisms for soil C sequestration in this N-rich forest.

First, elevated N addition decreased loss of soil C as CO_2_, possibly from both a negative priming effect (retarding microbial mineralization of soil organic C by altering microbial demand for nutrients) and a soil acidification effect. A negative priming effect could occur because, in high-N ecosystems, microbes no longer need to decompose recalcitrant organic matter to acquire N. Also, soil acidification induced by N addition (*SI Appendix*, Fig. S2*B*) can further drive negative priming effects at pH below 4.8 (28). This mechanism is supported by both the incubation experiment (29) and field monitoring (30) in this forest, where soil organic C mineralization and CO_2_ emission were declined greatly by elevated N addition, with lower soil pH suggested to be a driver. Soil pH may influence priming indirectly by altering the structure of the microbial community, which could slow C decomposition (28, 31). Declines in soil CO_2_ emissions, either through fertilization or through atmospheric N deposition (especially in sites receiving more than 50 kg N⋅ha^−1^⋅y^−1^) have been reported for temperate forest ecosystems as well (21). A substantial body of evidence suggests that anthropogenic N deposition can slow the microbial decay of plant detritus and potentially increase soil C storage across a wide range of terrestrial ecosystems (13, 15, 32).

**Fig. 2.**
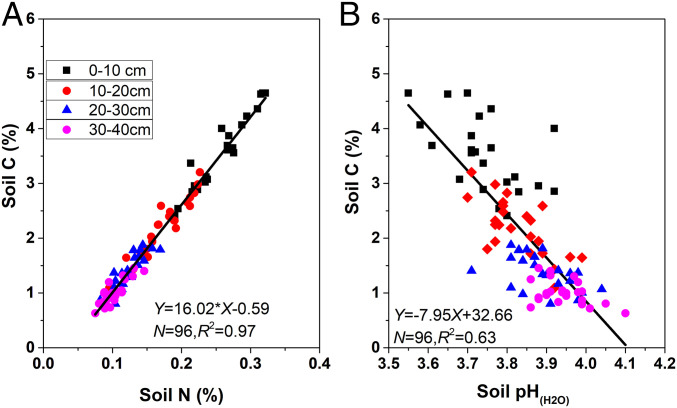
Relationships between soil C, N, and soil pH across all samples and plots at 0- to 40-cm soil layers in the N-saturated tropical forest of South China. (*A*) Soil C and N. (*B*) Soil C and soil pH. Note: Soils were sampled at 0- to 40-cm mineral layers after 11 y of N addition.

Second, elevated N addition decreased C leaching losses. A previous study at our site showed that N addition greatly decreased annual dissolved organic C (DOC) efflux below the rooting zone, and chemo-physical controls (solution acidity change and soil sorption) rather than biological controls mainly accounted for the decreased DOC efflux from this forest (33). In wet tropical soils, Lohse and Matson (34) suggested that chemical and physical mechanisms may be more important than biological processes in controlling N losses as well. Hagedorn et al. (35) concluded that the suppressed DOC fluxes from mineral soils under excess N inputs could be attributed to reduced mobilization of nonlitter-derived “older” DOC. Although DOC effluxes are smaller than C fluxes via soil respiration in terrestrial ecosystems, DOC mobilization and transport tightly link the bio-, hydro-, and pedosphere, playing an important role in soil forming processes and C storage. These mechanisms suggest that long-term N addition leads to greater soil organic carbon (SOC) stabilization, and that soil acidification could be an important contributor to soil C sequestration (28, 33), which is evidenced by the significant relationships between soil C and soil pH (Fig. 2*B*).

**Fig. 3.**
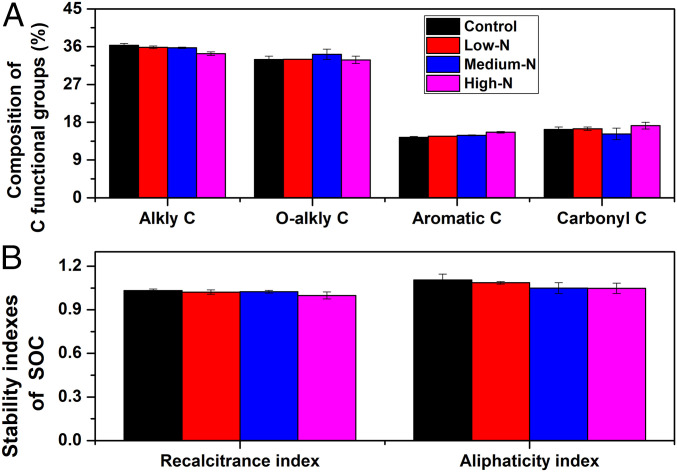
Effects of long-term N addition on soil organic C chemical compositions (*A*) and the stability indices of SOC (*B*) at the upper 0- to 10-cm layer in the N-saturated tropical forest at the Dinghushan reserve. Note: The stability indices include the recalcitrance index and the aliphaticity index. The recalcitrance index is the ratio of (alkyl + aromatic C)/(O-alkyl + carboxyl C). The aliphaticity index is the ratio of the alkyl region divided by the O-alkyl region. Nitrogen addition generally had no effects on the chemical composition and the stability indices of SOC.

We further found that long-term N addition significantly changed the physical composition of soil C; the heavy fraction C (organo-mineral associations) contributed to the elevated soil C storage, while the light fraction (physically unprotected particulate organic matter) decreased in percentage of bulk soil C (Table 1). Mineral-associated heavy fraction C typically has much longer turnover times than light fraction C (36), due to physical protection of soil C in highly weathered tropical soils (37). Evidence from sorption experiments has shown that organo-mineral associations generally reduce the susceptibility of organic matter to oxidation and the use by microbes (38). The fact that the majority of soil C pool at our site was dominated by the heavy fraction (∼80%) points to a major sink for C storage in soils and an increase in mineral-associated C pools under N treatments. However, elevated N addition did not change the chemical composition of soil C, such that the abundances of alkyl, O-alkyl, aromatic, and carbonyl C were generally not sensitive to N deposition (Fig. 3). The most plausible reason may be the lack of change in the biochemical composition of litter entering soil (e.g., C/N ratios; see *SI Appendix*, Table S1) and in microbial biomass (31). Using two decades of experimental N additions in northern hardwood forests, Zak et al. (15) found that biochemically equivalent organic matter has accumulated as heavy fraction C with mineral soil at a greater rate under experimental N deposition, relative to the ambient treatment. Physical interactions and occlusion between the surface of fine soil particles and organic matter decay products is the most plausible mechanism for increasing soil organic matter accumulation under N addition. If forests in the tropics respond in a manner similar to those in our experiment, then it is likely that the unabated deposition of anthropogenic N will increase the amounts and longevity of organic matter stored in their soils.

**Table 1.** Responses of soil carbon physical fractions to long-term N additions in the N-saturated tropical forest of South China

	Treatment	C stock, Mg C/ha	N stock, Mg N/ha	Percent of bulk soil C
Heavy fraction	Control	21.32 (1.02)^a^	1.71 (0.09)^a^	75.45 (2.28)^a^
	Low N	25.15 (1.39)^ab^	2.01 (0.07)^b^	79.30 (0.72)^ab^
	Medium N	28.07 (1.67)^b^	2.28 (0.07)^c^	81.23 (0.08)^b^
	High N	28.36 (1.90)^b^	2.25 (0.09)^bc^	82.97 (0.42)^b^
				
Light fraction	Control	6.91 (0.58)	0.30 (0.03)^a^	24.55 (2.28)^a^
	Low N	6.54 (0.18)	0.29 (0.00)^ab^	20.70 (0.72)^ab^
	Medium N	6.49 (0.38)	0.27 (0.01)^ab^	18.77 (0.08)^b^
	High N	5.82 (0.40)	0.26 (0.01)^b^	17.03 (0.42)^b^

Notes: Soils were sampled at the upper 0- to 10-cm mineral layers after 11 y of N addition. Values are means and SE (in parentheses); different letters indicate significant differences between treatments (*P* < 0.05).

### Soil C Sequestration and N Deposition in the Tropics.

**Fig. 4.**
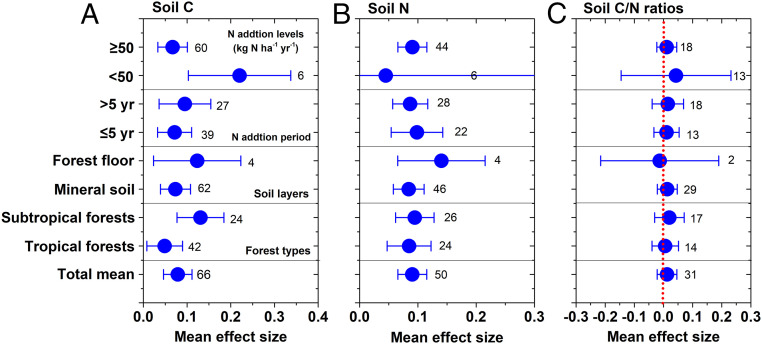
The mean effect sizes of experimental N addition on soil C (*A*), N (*B*), and C/N (*C*) ratios in the tropics. The variables are categorized into different groups depending on forest types, and N addition periods and levels. Error bars represent 95% confidence intervals (CIs). The dashed line was drawn at mean effect size of 0. The effect of N application was considered significant if the 95% CI of the effect size did not cover zero. The sample size for each variable is shown next to the point.

Although forests play a critical role in Earth’s terrestrial C sinks and exert strong control on atmospheric CO_2_ (24), the drivers of C sequestration, and the geographical extent and magnitude of this sink remain unclear. Our findings showed that long-term N input can contribute greatly to soil C sequestration in this primary tropical forest. This finding points to an urgent question—how general is this observation in the tropics, where atmospheric N deposition is projected to increase to much higher levels in 2050 as a result of intensifying industrial and agricultural activities (2). Hence, we explored a meta-analysis of N addition experiments to quantify the responses of soil C storage to N additions in tropical forests. Because of very limited data on soil C composition changes, we mainly focused on changes in total soil C storage. Our meta-analysis showed that N addition greatly increased forest soil C and N stocks (total mean by 8.2% and 9.5%, respectively) in both organic and mineral layers, across tropical zones and N application duration and levels (Fig. 4 *A* and *B*), demonstrating that chronic N deposition can simulate soil C sequestration in the tropical and subtropical forests as it did in our experimental site. Furthermore, elevated N inputs significantly accelerated soil acidification in the tropics where soil C stocks increased (Fig. 2*B* and *SI Appendix*, Fig. S3), suggesting that soil acidification could contribute to soil C accumulation (13, 15, 30). However, recent studies on tropical forest carbon budget, overwhelmingly focusing on aboveground measurements of gain and loss, showed that tropical forests made an approximately neutral or even negative contribution to the global carbon budget (24, 25) (*SI Appendix*, Table S2). Furthermore, new large-scale evidence that C uptake by intact tropical forests has already started a worrying downward trend since 1990s, which has consequences for policies intended to stabilize Earth’s climate (39). Therefore, soil C sink should be reconsidered in the undisturbed tropical forests, because organic matter absorbed to reactive soil minerals is an important mechanism for long-term C storage (40). In sum, tropical forest soils may be an underappreciated sink for CO_2_ with elevating atmospheric N deposition, although this sink will not be sufficient to compensate for all the C-emissions derived from fertilizer production, transport and application.

Interestingly, more than 5 y of high N addition (e.g., >50 kg N⋅ha^−1^⋅y^−1^) resulted in significant soil N retention in these tropical and subtropical forests (Fig. 4*B*), indicating a nonnegligible N retention capacity even in ecosystems with high N availability. Nitrogen can stabilize soil organic matter by forming recalcitrant associations with C, resulting in the accumulation of forms of stable organic matter over long periods of time (41). The nature of the C–N covalent bond in litter and soil organic matter can make N cycle more slowly, thus increasing N retention with increased soil C (42). We found no response of soil C/N ratios to variable N additions across both tropical and subtropical forests (Figs. 2*A* and 4*C* and *SI Appendix*, Fig. S2*C*), in contrast to other studies. In temperate forests, N inputs to N-limited ecosystems commonly increase soil C/N ratios from a stimulation in plant growth and subsequent high C input into soils (43). Investigating across (semi)natural ecosystems in Europe, Mulder et al. (44) found that soil C/N ratio was highly responsive to anthropogenic N deposition. Nitrogen-induced compositional changes in microbial communities have further functional implications for soil C cycling and storage in forest ecosystems (32, 45). Consistent C/N stoichiometry under variable N additions indicates a tight coupling of soil C and N, with soil N sequestration keeping pace with soil C in tropical ecosystems. This finding can contribute to predicting future sinks for both elevated anthropogenic CO_2_ and N deposition in the tropics.

### Soil C Sequestration Hypothesis.

Previous studies pointed to temperate and boreal forests as a predominant net global forest C sink, because gross emissions via tropical deforestation are mostly compensated by C uptake in both undisturbed and aggrading tropical forests (24). Our results demonstrate that this suggestion underestimates tropical forest C sinks, and that soils rather than woody tree biomass represent a significant C sink following long-term N addition in tropical forests. This study provides empirical evidence to address the long-debated question of whether mature tropical forests increase in soil C sequestration under elevated N deposition.

**Fig. 5.**
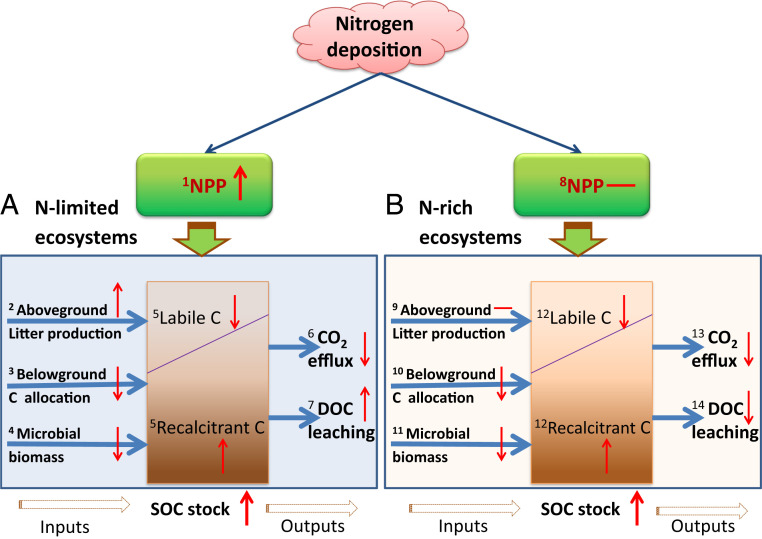
Soil C sequestration hypothesis: conceptual schemes on how soil C sequestration happens under chronic N deposition in forest ecosystems. (*A*) N-limited ecosystems. (*B*) N-rich ecosystems. Nitrogen addition-induced soil net C sequestration occurs mainly as a result of increased aboveground litter production and decreased CO_2_ effluxes in N-limited ecosystems, while decreased CO_2_ and DOC effluxes lead to much more soil C sequestration as recalcitrant forms of soil C under elevated N deposition in N-rich ecosystems. The hypothesis suggests that N deposition-induced soil C sequestration widely exists in terrestrial ecosystems, regardless of ecosystem N status and climate zones. Notes: The key ecological processes were marked with numbers; “↑” means increase, “↓” means decrease, and “—” means no response. NPP, net primary productivity.

To evaluate ecosystem C sequestration, we develop a conceptual model hypothesis depicting how soil C sequestration could occur under chronic N deposition in N-limited and N-rich ecosystems (Fig. 5). We suggest soil C sequestration has been happening with elevated N deposition at global scale, but the mechanisms are different between N-limited and N-rich ecosystems. In N-limited ecosystems (Fig. 5*A*), elevated N deposition generally increases NPP (7, 9, 11, 43), and subsequent aboveground litter production (9, 21, 22), but decreases below-ground C allocation (e.g., fine root biomass and turnover) and microbial biomass (13, 16, 21, 46), while CO_2_ efflux is decreased (13, 21) and leaching loss of DOC is increased (16, 47, 48). Nitrogen addition-induced soil net C sequestration occurs mainly as a result of increased aboveground litter production and decreased CO_2_ effluxes in N-limited ecosystems, which was supported by C isotopic evidence that N addition can stimulate soil C storage both by increasing soil C input and by decreasing decomposition rates (49), although N-induced enhancement of plant C inputs is not the primary mechanism in some cases (13). In N-rich ecosystems (Fig. 5*B*), however, chronic N deposition does not change NPP and aboveground litter production (this study, and see refs. 11 and 50), although below-ground C allocation and microbial biomass decreased greatly (46, 50). Decreased CO_2_ and DOC effluxes (30, 33, 50) lead to much more soil C sequestration as recalcitrant forms of soil C under elevated N deposition in N-rich ecosystems. As ecosystem C output, DOC leaching is the primary difference in SOC storage between the N-limited and N-rich ecosystems, which is supported by the meta-analysis at global scale, where elevated N addition significantly increased DOC effluxes in temperate and boreal forests, but decreased them in the tropical forests (*SI Appendix*, Supplementary Information Text and Fig. S4). Interestingly, N addition-induced stimulating effects on DOC leaching were weakened at a longer timescale (e.g., >5 y). Despite the distinct differences in key ecological processes involving C fluxes between two ecosystems, the patterns of soil C sequestration are similar, showing increased recalcitrant SOC but decreased labile SOC in both N-limited and N-rich ecosystems (Fig. 5 and ref. 21). Based on this hypothesis, we could predict that N deposition-induced soil C sequestration appears to be common in terrestrial forest ecosystems, regardless of ecosystem N status and climate zones (Fig. 4; also see refs. 16 and 21). The present scheme highlights a direction to incorporate N deposition and N cycling into terrestrial C cycle models to improve the predictability on C sink strength under global changes, because of the spread of enhanced N deposition from temperate into tropical systems (2).

## Methods

### Study Site.

This study was conducted at the DHSBR, which is a United Nations Educational, Scientific and Cultural Organization/Man and the Biosphere Programme site lying in the middle Guangdong Province, southern China (112°10′ E, 23°10′ N). The DHSBR has a monsoon climate and is in a subtropical/tropical moist forest life zone. The annual average precipitation is 1,748 mm from 2002 to 2012, mainly concentrated from April to September (26). Annual mean relative humidity is 80%. Mean annual temperature is 21.9 °C. The reserve has been experiencing high atmospheric N deposition in precipitation (e.g., commonly >30 kg N⋅ha^−1^⋅y^−1^) at least since the 1990s. In 2009 to 2010, total atmospheric N deposition was about 48.6 kg N⋅ha^−1^⋅y^−1^ with wet N deposition of 34.4 kg N⋅ha^−1^⋅y^−1^ (26).

We established our research site in 2002 in a primary monsoon evergreen broadleaf forest (climax forest) between 250 and 300 m above sea level. The forest has been protected from disturbances related to land use for >400 y (51), and supports a rich assemblage of plant species, most of which are distributed throughout tropical and subtropical China, including *Castanopsis chinensis* Hance, *Schima superba* Chardn. and Champ., *Cryptocarya chinensis* (Hance) Hemsl., *Machilus chinensis* (Champ. Ex Benth.) Hemsl., *Syzygium rehderianum* Merr. and Perry, and *Acmena acuminatissima* (Blume) Merr. et Perry. Canopy closure is typically above 95% (52). Soils in the study site are lateritic red earths (Oxisols) formed from sandstone with a common depth of 40 cm (ranging from 30 to 70 cm), and are highly weathered with poor soil-buffering capacity (53). The primary forest has been N-saturated (30, 52, 53), and is an N-rich ecosystem as are many lowland tropical forests (26).

### Experimental Treatments.

Nitrogen amendments were initiated in July 2003, with four rates used: control (0 N added), low-N (50 kg N⋅ha^−1^⋅y^−1^), medium-N (100 kg N⋅ha^−1^⋅y^−1^), and high-N (150 kg N⋅ha^−1^⋅y^−1^). These were based on present atmospheric N deposition rate and the expected increase in the future due to the rapid development of agricultural and industrial activities (54, 55). In total, there were 12 10- × 20-m plots surrounded by buffer strips that were at least 20 m in width, with treatments replicated in triplicate and randomly assigned. Monthly applications of NH_4_NO_3_ solution were sprayed onto the plots at constant rates throughout the year. The control treatment consisted of equal volumes (20 L) of deionized water.

### Field Sampling and Laboratory Analysis.

Soils at the upper 0- to 10-cm layers were randomly collected during the summer seasons (July to September) in the years of 2003, 2004, 2006, 2009, and 2014, respectively. The cores were taken beneath the loose litter layer (Oi) and were composed of Oe and Oa horizon plus mineral soil to a total depth of 10 cm. In 2014, we enriched soil sampling, including forest floor layers and deeper mineral soil layers (0 to 40 cm). Forest floor layer (organic horizon), which mainly consists of undecomposed plant materials on the soil surface (mainly as Oi layer), was collected at two random points on an area basis using a 20 × 20-cm wooden frame. Mineral soil samples were taken using an auger (5.1 cm in diameter) and divided into four layers (0 to 10, 10 to 20, 20 to 30, and 30 to 40 cm). Two soil cores were taken from each plot and analyzed separately but only plot mean values were used. In the laboratory, soils were sieved (2 mm) to remove roots and stones, and mixed thoroughly by hand for subsequent chemical analysis. In addition, litterfall in each plot was collected monthly during July 2013 and June 2014 using two litter traps (1 × 1 m) with 1-mm mesh in each plot at 1-m height. Litterfall was separated into leaves and others (branches, fruits, flowers, barks).

All litters and soil subsamples were oven-dried at 70 °C and ground to a fine and homogeneous powder. Carbon, N, and ^13^C contents of all samples were determined simultaneously on an isotope ratio mass spectrometer (Isoprime 100; Isoprime) coupled to an automatic, online elemental analyzer (vario ISOTOPE cube).

Soil C fractions were measured for soils sampled in 2014. Soil organic matter was separated by density into light fraction and heavy fraction using a modification method based on the procedures of Janzen et al. (56) and Strickland and Sollins (57). The light fractions with low density (<1.7 g⋅cm^−3^) are partly decayed organic residues, representing physically unprotected SOM pool, turning over on short timescales; while heavy fractions with high density (>1.7 g⋅cm^−3^) referred to humic substance, which are generally mineral associated, representing physically stabilized SOM and turning over on longer timescales. Specifically, 10 g of air-dried soil was placed in a centrifuge tube and added to 50 mL of NaI solution with a density of 1.7 g⋅cm^−3^. The tubes were shaken on a shaker for 60 min with a frequency of 250 per min, and then centrifuged at 1,000 rpm for 10 min. The floating light fraction was sucked on a 0.45-μm polycarbonate filter. This process was repeated twice in order to separate the light and heavy fractions totally. After that, the material remaining at the bottom of the tube (the heavy fraction) was added to 50 mL of deionized water, shaken and centrifuged for three times to wash. The light fraction was rinsed with 100 mL of 0.01 M CaCl_2_ and then 100 mL of deionized water until all NaI was removed. Both the light fraction and heavy fraction were dried at 60 °C for 48 h, weighed, and ground to determine the C and N contents as described above.

To explore how long-term N additions affect the chemical composition of soil organic matter, we resampled the top 0- to 10-cm soils in 2017. Approximately 5 g was weighted into 100-mL polyethylene centrifuge tube and shaken end-over-end with 8 (4*1 h, 3*12 h, and 1* 24 h) successive 50 mL of 10% hydrofluoric acid (vol/vol). Samples were centrifuged after each extraction at 3,000 rpm for 10 min, and the supernatant was removed with a tube attached to a plastic syringe to prevent the loss of fine material by decanting. The remaining sediment was washed with distilled water and over dried at 40 °C, and ground to a powder for further analysis. We employed solid-state ^13^C-NMR spectroscopy to determine the relative abundance of alkyl C, O-alkyl C, aromatic C, and carboxyl C, after demineralization with 10% hydrofluoric acid (to remove mineral material including paramagnetic compounds such as Fe and to concentrate the SOM). The spectra were recorded on a Bruker AV-300 NMR spectrometer at a frequency of 75.5 MHz. Cross-polarization magic-angle-spinning (CPMAS) was applied at 12 kHz, with contact time of 35 ms, recycle time of 5 s, and zero-filled of 2,000 data points. To quantify the different types of C, the CPMAS ^13^C NMR spectra were divided into four chemical shift regions: alkyl C (0 to 50 ppm), O-alkyl C (50 to 110 ppm), aromatic C (110 to 160 ppm), and carboxyl C (160 to 220 ppm). The integration of the peaks within each of the chemical shift regions allowed us to estimate the relative C contents, expressed as the percent ratio of the peak area of these groups to the total peak area in each composite sample. We further calculated the recalcitrance index and the aliphaticity index, which were related to the stability of SOC. The recalcitrance index is the ratio of (alkyl + aromatic C)/(O-alkyl + carboxyl C). Alkyl and aromatic compounds include long-chain aliphatics and tannin, among other compounds, which are hydrophobic and resist decay, whereas O-alkyl and carboxyl C represent compounds such as organic acids, which are hydrophilic and labile. The aliphaticity index is the ratio of the alkyl region divided by the O-alkyl region. A higher ratio signifies a greater contribution of alkyl C, such as lipids and other aliphatic compounds. A lower ratio indicates a greater contribution of O-alkyl C, mostly represented by plant carbohydrates, including cellulose and hemicellulose, which are considered more labile than alkyl C to microbial decomposers.

Soil for bulk density was randomly collected from undisturbed soil within each plot in 2014, using a stainless-steel core (5.65 cm in diameter, 4 cm in depth, and 100 cm^3^ in volume). We used these soil bulk densities to calculate soil C storage. Soil C storage is the product of soil C concentration, layer thickness, and bulk density.

### Data Compilation.

We searched the peer-reviewed journal articles on March 31, 2018 in Web of Science (1900 to 2017), using the following search criteria: “forest” and “carbon” and “nitrogen addition or nitrogen deposition or nitrogen enrichment or nitrogen fertilization.” This search returned 5,405 papers, the titles of which were scanned to eliminate obviously irrelevant papers, resulting in a list of 110 candidate papers. Candidate papers were individually examined for data meeting the following criteria (*SI Appendix*, Table S3):

1) To avoid possible confounding factors caused by site conditions, we only included studies in which control and N addition treatment sites experienced the same climatic, soil, and vegetation conditions. Studies along N deposition gradients were excluded.
2) Measurements from different N addition levels, N addition types, soil depths, or ecosystems within a single study were considered as independent. For several long-term research sites, to meet the statistical assumption of independence among observations, only the latest results were included for multiple measurements over time.
3) For studies that examined the interactions of N addition with other global change factors (e.g., elevated CO_2_, climate warming, changing precipitation, etc.), only data from the control and N addition plots were included, because additional factors can interact with N inputs and affect soil C and N cycling in complex ways.
4) Only field studies performed in forest ecosystems were used, and plot areas should be ≥10 m*10 m.
5) Means, SDs (or SEs), and numbers of replication were directly provided or could be calculated.

When data were graphically presented, figures were digitized to extract the numerical values using Engauge Digitizer (Free Software Foundation). Soil pH values were also extracted if available. We selected data for the tropical and subtropical forest ecosystems, including tropical forests (0 to 23.5° S/N) and subtropical forests (23.5° to 30° S/N).

### Statistical Analyses.

Repeated-measure ANOVA was performed to examine the overall effects of N treatments on soil C and N dynamics during the study period. One-way ANOVA with Fisher LSD (least-significant difference) multiple range test was performed to determine the effects of N treatment on soil/litter C, N, and C/N ratios, soil pH, litter mass, soil C physical fractions, and chemical compositions. We conducted the planned contrast analysis to test differences between control plots and N treatment plots. A general linear model was used to analyze the relationships between soil C and N, and soil pH across all samples and plots. All analyses were conducted using SPSS 14.0 for Windows (SPSS). Statistically significant differences were set with values of *P* < 0.05, unless otherwise stated. All data met the assumptions of the tests.

For meta-analysis, we calculated the mean effect size and 95% CI of the overall effects of experimental N additions on soil C and N contents, C/N ratios, and soil pH, using the methods described by Hedges et al. (58). The effects of N additions were estimated based on the natural log-transformed response ratio (RR):

$$\ln\mathrm{RR}=\ln\left({\bar{X}}_{t}/{\bar{X}}_{c}\right),$$

where ${\bar{X}}_{t}$ is the treatment mean, and ${\bar{X}}_{c}$ is the control mean. The variance (*v*) associated with each value of lnRR was calculated as follows:

$$\nu =\frac{s_{t}^{2}}{n_{t}{\bar{x}}_{t}^{2}}\;+\;\frac{s_{c}^{2}}{n_{c}{\bar{x}}_{c}^{2}},$$

where $n_{t}$ and $n_{c}$ are the sample sizes for the treatment and control groups, respectively; and $s_{t}$ and $s_{c}$ are the SDs for the treatment and control groups, respectively.

The mean response ratio ($\bar{\ln\mathrm{RR}}$) was calculated using a fixed-effects model of the meta-analytical software, METAWIN (Sinauer Associates). Confidence intervals (CIs) on the weighted effect size were generated using bootstrapping (9,999 iterations). If the 95% CI values of the effect size for a variable did not overlap with 0, the effect of N additions on the variable was considered to differ significantly between two treatments. Because N addition did not cause statistically significant differences in soil bulk density, response ratios of soil C contents in response to N applications were used to represent changes in soil C pool sizes (18).

## Supplementary Material

Supplementary File

## Data Availability

Data have been deposited with the South China Botanical Garden of the Chinese Academy of Sciences (https://u.bgarden.net/f/e723a52526e9445798f0/) All other study data are included in the article and/or *SI Appendix*.

## References

[1] P. A. Matson, W. H. McDowell, A. R. Townsend, P. M. Vitousek, The globalization of N deposition: Ecosystem consequences in tropical environments. Biogeochemistry 46, 67–83 (1999).

[2] J. N. Galloway., Nitrogen cycles: Past, present, and future. Biogeochemistry 70, 153–226 (2004).

[3] D. S. Reay., Global nitrogen deposition and carbon sinks. Nat. Geosci. 1, 430–437 (2008).

[4] P. M. Vitousek, R. W. Howarth, Nitrogen limitation on land and in the seas: How can it occur? Biogeochemistry 13, 87–115 (1991).

[5] D. S. LeBauer, K. K. Treseder, Nitrogen limitation of net primary productivity in terrestrial ecosystems is globally distributed. Ecology 89, 371–379 (2008). 18409427 10.1890/06-2057.1

[6] A. R. Townsend, B. H. Braswell, E. A. Holland, J. E. Penner, Spatial and temporal patterns in terrestrial carbon storage due to deposition of fossil fuel nitrogen. Ecol. Appl. 6, 806–814 (1996).

[7] F. Magnani., The human footprint in the carbon cycle of temperate and boreal forests. Nature 447, 848–850 (2007). 17568744 10.1038/nature05847

[8] R. Hyvönen., The likely impact of elevated [CO_2_], nitrogen deposition, increased temperature and management on carbon sequestration in temperate and boreal forest ecosystems: A literature review. New Phytol. 173, 463–480 (2007). 17244042 10.1111/j.1469-8137.2007.01967.x

[9] R. Q. Thomas, C. D. Canham, K. C. Weathers, C. L. Goodale, Increased tree carbon storage in response to nitrogen deposition in the US. Nat. Geosci. 3, 13–17 (2010).

[10] W. de Vries, E. Du, K. Butterbach-Bahl, Short and long-term impacts of nitrogen deposition on carbon sequestration by forest ecosystems. Curr. Opin. Environ. Sustainability 9–10, 90–104 (2014).

[11] L. Schulte-Uebbing, W. de Vries, Global-scale impacts of nitrogen deposition on tree carbon sequestration in tropical, temperate, and boreal forests: A meta-analysis. Glob. Change Biol. 24, e416–e431 (2018). 10.1111/gcb.13862 29034987

[12] W. H. Schlesinger, E. S. Bernhardt, Biogeochemistry: An Analysis of Global Change (Academic Press, 2013).

[13] S. D. Frey., Chronic nitrogen additions suppress decomposition and sequester soil carbon in temperate forests. Biogeochemistry 121, 305–316 (2014).

[14] G. M. Lovett, M. A. Arthur, K. C. Weathers, R. D. Fitzhugh, P. H. Templer, Nitrogen addition increases carbon storage in soils, but not in trees, in an eastern U.S. deciduous forest. Ecosystems (N. Y.) 6, 980–1001 (2013).

[15] D. R. Zak, Z. B. Freedman, R. A. Upchurch, M. Steffens, I. Kögel-Knabner, Anthropogenic N deposition increases soil organic matter accumulation without altering its biochemical composition. Glob. Change Biol. 23, 933–944 (2017). 10.1111/gcb.13480 27562874

[16] L. Liu, T. L. Greaver, A global perspective on belowground carbon dynamics under nitrogen enrichment. Ecol. Lett. 13, 819–828 (2010). 20482580 10.1111/j.1461-0248.2010.01482.x

[17] J. L. DeForest, D. R. Zak, K. S. Pregitzer, A. J. Burton, Atmospheric nitrate deposition and the microbial degradation of cellobiose and vanillin in a northern hardwood forest. Soil Biol. Biochem. 36, 965–971 (2004).

[18] M. Lu., Minor stimulation of soil carbon storage by nitrogen addition: A meta-analysis. Agric. Ecosyst. Environ. 140, 234–244 (2011).

[19] H. Van Miegroet, R. Jandl, Are nitrogen-fertilized forest soils sinks or sources of carbon? Environ. Monit. Assess. 128, 121–131 (2007). 17180428 10.1007/s10661-006-9410-7

[20] S. J. Forstner., Vertical redistribution of soil organic carbon pools after twenty years of nitrogen addition in two temperate coniferous forests. Ecosystems (N. Y.) 22, 379–400 (2019). 10.1007/s10021-018-0275-8 PMC6423314 30956544

[21] I. A. Janssens., Reduction of forest soil respiration in response to nitrogen deposition. Nat. Geosci. 3, 315–322 (2010).

[22] W. de Vries., The impact of nitrogen deposition on carbon sequestration by European forests and heathlands. For. Ecol. Manage. 258, 1814–1823 (2009).

[23] E. N. J. Brookshire, S. Gerber, D. N. Menge, L. O. Hedin, Large losses of inorganic nitrogen from tropical rainforests suggest a lack of nitrogen limitation. Ecol. Lett. 15, 9–16 (2012). 22017659 10.1111/j.1461-0248.2011.01701.x

[24] Y. Pan., A large and persistent carbon sink in the world’s forests. Science 333, 988–993 (2011). 21764754 10.1126/science.1201609

[25] A. Baccini., Tropical forests are a net carbon source based on aboveground measurements of gain and loss. Science 358, 230–234 (2017). 28971966 10.1126/science.aam5962

[26] X. Lu., Plant acclimation to long-term high nitrogen deposition in an N-rich tropical forest. Proc. Natl. Acad. Sci. U.S.A. 115, 5187–5192 (2018). 29717039 10.1073/pnas.1720777115 PMC5960300

[27] R. Lal, J. M. Kimble, R. F. Follett, B. A. Stewart, Soil Processes and the Carbon Cycle (CRC Press, Boca Raton, FL, 1997).

[28] A. T. Nottingham, B. L. Turner, A. W. Stott, E. V. Tanner, Nitrogen and phosphorus constrain labile and stable carbon turnover in lowland tropical forest soils. Soil Biol. Biochem. 80, 26–33 (2015).

[29] X. Ouyang., Effect of N and P addition on soil organic C potential mineralization in forest soils in South China. J. Environ. Sci. (China) 20, 1082–1089 (2008). 19143315 10.1016/s1001-0742(08)62153-1

[30] J. Mo., Nitrogen addition reduces soil respiration in a mature tropical forest in southern China. Glob. Change Biol. 14, 403–412 (2008).

[31] C. Wang., Responses of soil microbial community to continuous experimental nitrogen additions for 13 years in a nitrogen-rich tropical forest. Soil Biol. Biochem. 121, 103–112 (2018).

[32] F. S. Gilliam., Decreased atmospheric nitrogen deposition in eastern North America: Predicted responses of forest ecosystems. Environ. Pollut. 244, 560–574 (2019). 30384062 10.1016/j.envpol.2018.09.135

[33] X. Lu, F. S. Gilliam, G. Yu, H. Chen, J. Mo, Long-term nitrogen addition decreases carbon transport in nitrogen-rich forest. Biogeosciences 10, 1451–1481 (2013).

[34] K. A. Lohse, P. Matson, Consequences of nitrogen additions for soil processes and soil solution losses from wet tropical forests. Ecol. Appl. 15, 1629–1648 (2005).

[35] F. Hagedorn, A. Kammer, M. W. I. Schmidt, C. L. Goodale, Nitrogen addition alters mineralization dynamics of ^13^C-depleted leaf and twig litter and reduces leaching of older DOC from mineral soil. Glob. Change Biol. 18, 1412–1427 (2012).

[36] J. D. Hemingway., Mineral protection regulates long-term global preservation of natural organic carbon. Nature 570, 228–231 (2019). 31190013 10.1038/s41586-019-1280-6

[37] E. Marin-Spiotta, W. L. Silver, C. W. Swanston, R. Ostertag, Soil organic matter dynamics during 80 years of reforestation of tropical pastures. Glob. Change Biol. 15, 1584–1597 (2009).

[38] I. Kögel-Knabner., Organo-mineral associations in temperate soils: Integrating biology, mineralogy, and organic matter chemistry. J. Plant Nutr. Soil Sci. 171, 61–82 (2008).

[39] W. Hubau., Asynchronous carbon sink saturation in African and Amazonian tropical forests. Nature 579, 80–87 (2020). 32132693 10.1038/s41586-020-2035-0 PMC7617213

[40] M. G. Kramer, O. A. Chadwick, Climate-driven thresholds in reactive mineral retention of soil carbon at the global scale. Nat. Clim. Chang. 8, 1104–1108 (2018).

[41] D. W. Johnson, J. Turner, Nitrogen budgets of forest ecosystems: A review. For. Ecol. Manage. 318, 370–379 (2014).

[42] P. M. Vitousek, S. Hättenschwiler, L. Olander, S. Allison, Nitrogen and nature. Ambio 31, 97–101 (2002). 12078015 10.1579/0044-7447-31.2.97

[43] J. Aber., Nitrogen saturation in temperate forest ecosystems. Bioscience 48, 921–934 (1998).

[44] C. Mulder., Chemical footprints of anthropogenic nitrogen deposition on recent soil C:N ratios in Europe. Biogeosciences 12, 4113–4119 (2015).

[45] S. D. Eisenlord., Microbial mechanisms mediating increased soil C storage under elevated atmospheric N deposition. Appl. Environ. Microbiol. 79, 1191–1199 (2013). 23220961 10.1128/AEM.03156-12 PMC3568582

[46] Y. Peng, D. Guo, Y. Yang, Global patterns of root dynamics under nitrogen enrichment. Glob. Ecol. Biogeogr. 26, 102–114 (2017).

[47] K. S. Pregitzer, D. R. Zak, A. J. Burton, J. A. Ashby, N. W. MacDonald, Chronic nitrate additions dramatically increase the export of carbon and nitrogen from northern hardwood ecosystems. Biogeochemistry 68, 179–197 (2004).

[48] S. E. Findlay, Increased carbon transport in the Hudson River: Unexpected consequence of nitrogen deposition? Front. Ecol. Environ. 3, 133–137 (2005).

[49] X. Huang., New soil carbon sequestration with nitrogen enrichment: A meta-analysis. Plant Soil 454, 299–310 (2020).

[50] D. F. Cusack, W. L. Silver, M. S. Torn, W. H. McDowell, Effects of nitrogen additions on above-and belowground carbon dynamics in two tropical forests. Biogeochemistry 104, 203–225 (2011).

[51] C. Shen., ^14^C measurement of forest soils in Dinghushan Biosphere Reserve. Chin. Sci. Bull. 44, 251–256 (1999).

[52] X. Lu., Effects of experimental nitrogen additions on plant diversity in an old‐growth tropical forest. Glob. Change Biol. 16, 2688–2700 (2010).

[53] X. Lu, Q. Mao, F. S. Gilliam, Y. Luo, J. Mo, Nitrogen deposition contributes to soil acidification in tropical ecosystems. Glob. Change Biol. 20, 3790–3801 (2014). 10.1111/gcb.12665 24953639

[54] C. Lü, H. Tian, Spatial and temporal patterns of nitrogen deposition in China: Synthesis of observational data. J. Geophys. Res. D Atmospheres 112, D22S05 (2007).

[55] N. Gruber, J. N. Galloway, An Earth-system perspective of the global nitrogen cycle. Nature 451, 293–296 (2008). 18202647 10.1038/nature06592

[56] H. H. Janzen, C. A. Campbell, S. A. Brandt, G. P. Lafond, L. Townley-Smith, Light-fraction organic matter in soils from long-term crop rotations. Soil Sci. Soc. Am. J. 56, 1799–1806 (1992).

[57] T. C. Strickland, P. Sollins, Improved method for separating light-and heavy-fraction organic material from soil. Soil Sci. Soc. Am. J. 51, 1390–1393 (1987).

[58] L. V. Hedges, J. Gurevitch, P. S. Curtis, The meta‐analysis of response ratios in experimental ecology. Ecology 80, 1150–1156 (1999).

