# Supplementary material for: Nitrogen deposition accelerates soil carbon sequestration in tropical forests
Source: Proc Natl Acad Sci U S A. 2021 Apr 12;118(16):e2020790118. doi: 10.1073/pnas.2020790118 (PMC8072245; doi:10.1073/pnas.2020790118)
Supplement: Supplementary File [file pnas.2020790118.sapp.pdf]

PNAS
www.pnas.org

Supplementary Information for

Nitrogen deposition accelerates soil carbon sequestration in tropical forests

Xiankai Lu[1*], Peter M. Vitousek[2*], Qinggong Mao[1], Frank S. Gilliam[3], Yiqi Luo[4], Benjamin L. Turner[5], Guoyi Zhou[1], Jiangming Mo[1*]

*Corresponding author: Xiankai Lu (luxiankai@scbg.ac.cn), Peter M. Vitousek (vitousek@stanford.edu), or Jiangming Mo (mojm@scib.ac.cn).

This PDF file includes:

Supplementary text
Figures S1 to S3
Tables S1 to S3

Supplementary Information Text

Data compilation for DOC. To explore the responses of dissolved organic carbon (DOC) effluxes to elevated N addition in forest ecosystems at global scale, we searched the peer-reviewed journal articles on 21 November 2020 in Web of Science® (1900-2020), using the following search criteria: "forest" and "DOC or DOM or dissolved organic carbon or dissolved organic matter" and "nitrogen addition or nitrogen deposition or nitrogen enrichment or nitrogen fertilization". This search returned 1,266 papers, the titles of which were scanned to eliminate obviously irrelevant papers, resulting in a list of 52 candidate papers. Candidate papers were individually examined for data meeting the criteria same to the data compilation for soil C and N contents in the main text. We selected data for the tropical and extra-tropical forest ecosystems, including tropical forests (0-23.5° S/N), subtropical forests (23.5°-30° S/N), temperate forests (30°-50° S/N) and boreal forests (50°-60° S/N). The related references are listed as following.

(1) Z. Lei, Q. Li, X. Song, W. Wang, Z. Zhang, C. Peng, L. Tian, Biochar mitigates dissolved organic carbon loss but does not affect dissolved organic nitrogen leaching loss caused by nitrogen deposition in Moso bamboo plantations. *Global Ecology and Conservation* 16, art. no. e00494 (2018).

(2) P. Oulehle, et al. Comparison of the impacts of acid and nitrogen additions on carbon fluxes in European conifer and broadleaf forests. *Environmental Pollution* ***238***, 884-893 (2018).

(3) A. Velescu, C. Valarezo, W. Wilcke, Response of Dissolved Carbon and Nitrogen Concentrations to Moderate Nutrient Additions in a Tropical Montane Forest of South Ecuador. *Front. Earth Sci*. 4, 58 (2016).

(4) G.M. Lovett, M.A. Arthur, K.C. Weathers, et al., Nitrogen Addition Increases Carbon Storage in Soils, But Not in Trees, in an Eastern U.S. Deciduous Forest. *Ecosystems* 16, 980–1001 (2013)

(5) L. Duan, X. Ma, D. Yu, B. Tan, Effects of simulated nitrogen deposition on organic matter leaching in forest soil. *Environmental Science* 34(6):2422-2427 (2013).

(6) M. Fröberg, H. Grip,E. Tipping, et al., Long-term effects of experimental fertilization and soil warming on dissolved organic matter leaching from a spruce forest in Northern Sweden. *Geoderma* 200-201:172-179 (2013).

(7) X. Lu, F. S. Gilliam, G. Yu, L. Li, Q. Mao, H. Chen, J. Mo, Long-term nitrogen addition decreases carbon leaching in a nitrogen-rich forest ecosystem, *Biogeosciences* 10, 3931–3941 (2013)

(8) M.O. Rappe-George, A.I. Gärdenäs, D. B. Kleja, The impact of four decades of annual nitrogen addition on dissolved organic matter in a boreal forest soil, *Biogeosciences* 10, 1365–1377 (2013).

(9) F.R. Fatemi, I.J. Fernandez, J. Szillery, S.A. Norton, L.E. Rustad, Soil Solution Chemical Response to Two Decades of Experimental Acidification at the Bear Brook Watershed in Maine. *Water, Air, and Soil Pollution* 223(9), 6171-6186 (2012).

(10) J.S. Szillery, Soil solution dynamics in response to elevated nitrogen and sulfur treatments at the Bear Brook Watershed in Maine. doi:http://worldcat.org/ oclc/62275814 (2003)

(11) D.F. Cusack, W.L. Silver, M.S. Torn, et al*.,* Effects of nitrogen additions on above- and belowground carbon dynamics in two tropical forests. *Biogeochemistry* 104, 203–225 (2011).

(12) Y. Fang, W. Zhu, P. Gundersen, et al*.,* Large Loss of Dissolved Organic Nitrogen from Nitrogen-Saturated Forests in Subtropical China. *Ecosystems* 12, 33–45 (2009).

(13) C.D. Evans, C.L. Goodale, S.J.M. Caporn, et al., Does elevated nitrogen deposition or ecosystem recovery from acidification drive increased dissolved organic carbon loss from upland soil? A review of evidence from field nitrogen addition experiments. *Biogeochemistry* 91, 13-35 (2008)

(14) K.A. Smemo, et al., Characteristics of DOC exported from northern hardwood forests receiving chronic experimental NO_3^- deposition. *Ecosystems* 10(3), 369-379 (2007).

(15) A.H. Magill, J. D. Aber, W. S. Currie, et al., Ecosystem response to 15 years of chronic nitrogen

additions at the Harvard Forest LTER, Massachusetts, USA. *For Ecol Manag* 196, 7–28 (2004).
(16)W. H. Mcdowell, A. H. Magill, J. A. Aitkenhead-Peterson, et al., Effects of chronic nitrogen amendment on dissolved organic matter and inorganic nitrogen in soil solution. *For Ecol Manag* 196, 29–41(2004).
(17)K.S. Pregitzer, D.R. Zak, A.J. Burton, J.A. Ashby, N.W. MacDonald, Chronic nitrate additions dramatically increase the export of carbon and nitrogen from northern hardwood ecosystems. *Biogeochemistry* **68**, 179–197 (2004).
(18)Y. Yano, W.H. McDowell, J.D. Aber, Biodegradable dissolved organic carbon in forest soil solution and effects of chronic nitrogen deposition. *Soil Biol Biochem* 32, 1743–1751(2000).
(19)A.H. Magill, J.D. Aber, G.M. Berntson, et al., Long-Term Nitrogen Additions and Nitrogen Saturation in Two Temperate Forests. *Ecosystems* 3(3), 238-253 (2000).
(20)S. Norton, J. Kahl, I. Fernandez, et al., The Bear Brook Watershed, Maine USA (BBWM). *Environ Monit Assess* **55**, 7–51(1999).
(21)B.A. Emmett, B. Reynolds, M. Silgram, T.H. Sparks, C. Woods, The consequences of chronic nitrogen additions on N cycling and soilwater chemistry in a Sitka spruce stand, North Wales. *For Ecol Manag* **101**,165–175 (1998)
(22)W.S. Currie, J.D. Aber, W.H. Mcdowell, et al*.* Vertical transport of dissolved organic C and N under long-term N amendments in pine and hardwood forests. *Biogeochemistry* **35**(3), 471-505 (1996).

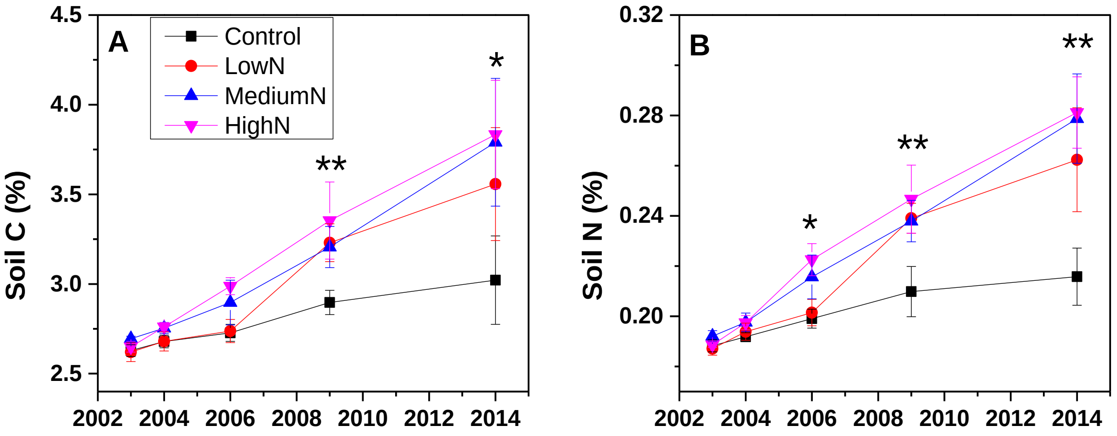

Fig. S1 Long-term dynamics of total soil C (A) and N concentrations (B) at top 0-10 cm soil layer under N treatments in the N-rich tropical forest of South China. Note: Total soil C and N concentrations were comparable between treatments at the beginning of N additions in July 2003. Repeated measures ANOVA showed that long-term N addition significantly increased soil C and N concentrations (P<0.01); Asterisks * and ** indicate that there are significant differences between N treatments and the Controls at P < 0.1and P<0.05 levels, respectively; Values are means with S.E.

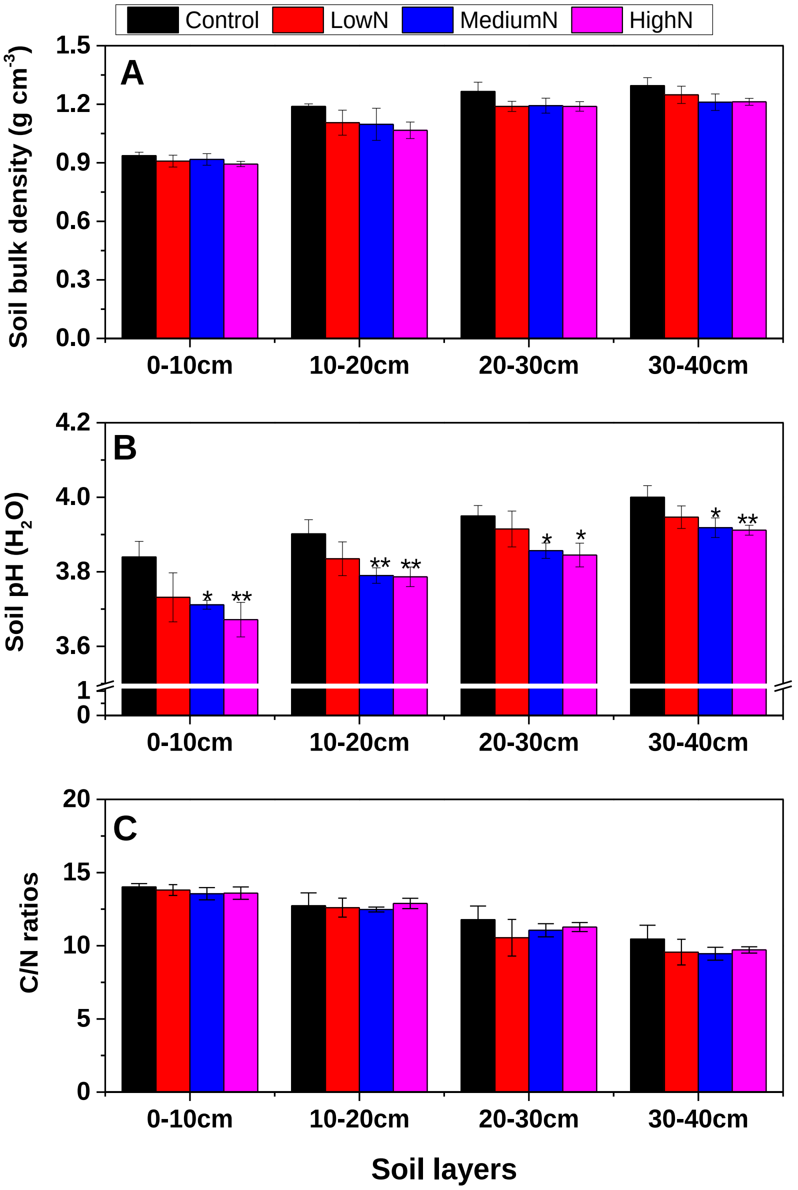

Fig. S2 Effects of long-term N additions on soil bulk density (a), soil pH (b), and soil C/N ratios at 0-40cm soil layers in the N-rich tropical forest at the Dinghushan reserve. Note: Soil were sampling after 11 years of N addition; Asterisks ***** and ****** indicate that there are significant differences between N treatments and the Controls at P < 0.1 and P<0.05 levels, respectively; Values are means with S.E.

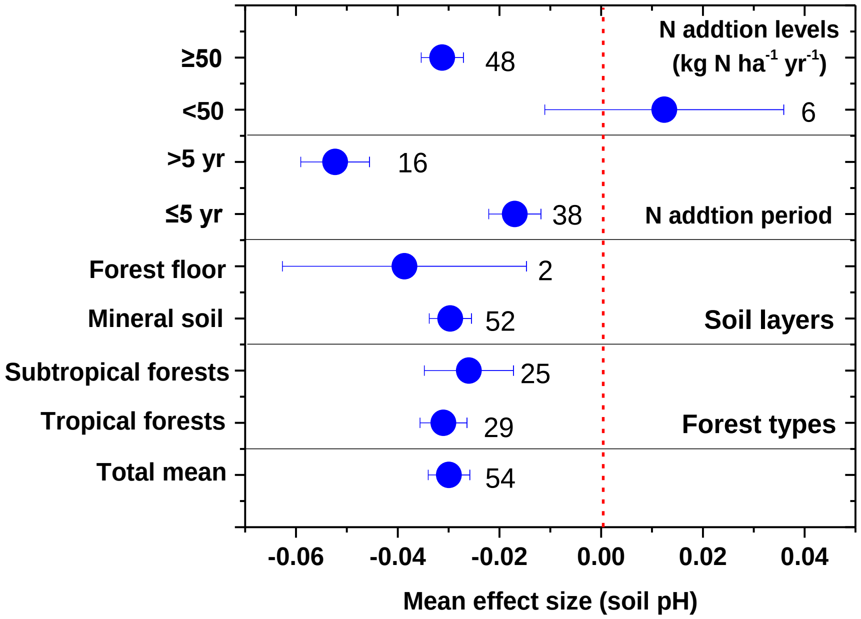

Fig. S3 The mean effect sizes of experimental N additions on soil pH in the tropics. The variables are categorized into different groups depending on forest types and soil layers. Nitrogen additions significantly decreased soil pH values in both mineral soil layers (by 3.2 % over the controls) and forest floor (by -0.8 %), and in all forest types. Error bars represent 95% confidence intervals (CIs). The dashed line was drawn at mean effect size = 0. The effect of N application was considered significant if the 95% CI of the effect size did not cover zero. The sample size for each variable is shown next to the point. The papers for data compilation are same to those of soil C and N contents (see Table S3).

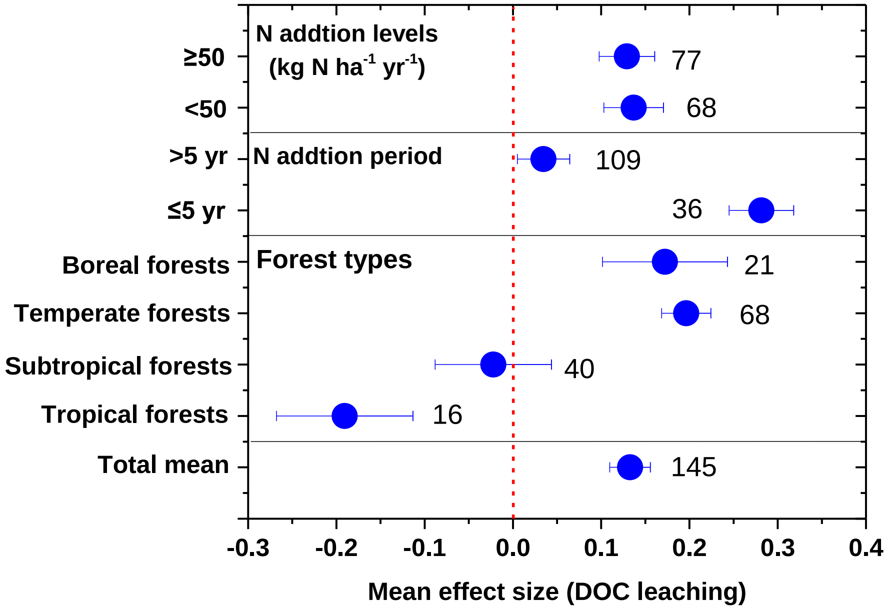

Fig. S4 The mean effect sizes of experimental N addition on soil dissolved organic carbon (DOC) effluxes in forest ecosystems at the global scale. The variables are categorized into different groups depending on forest types, and N addition periods and levels. Error bars represent 95% confidence intervals (CIs). The dashed line was drawn at mean effect size = 0. The effect of N application was considered significant if the 95% CI of the effect size did not cover zero. The sample size for each variable is shown next to the point. The meta-analysis method is shown in Supplementary Information Text.

Table S1 Responses of annual fresh foliar litterfall and forest floor layer to long-term N additions in the N-saturated tropical forest at the Dinghushan reserve. Notes: Foliar litterfall was collected during July 2013 and June 2014, and forest floor layer (organic layer) was sampled in July 2014 (after 11 years of N additions). Values are means and S.E (in parenthesis).

Treatments	Mass (Mg/ha)	C stock (Mg C/ha)	N stock (Mg N/ha)	C/N
Fresh foliar litterfall				
Control	3.81(0.14)	1.77(0.06)	0.06(0.00)	28.94(1.42)
Low-N	3.44(0.20)	1.60(0.09)	0.05(0.00)	31.33(0.40)
Medium-N	3.84(0.43)	1.81(0.20)	0.06(0.01)	29.78(0.88)
High-N	3.24(0.26)	1.52(0.13)	0.05(0.00)	29.36(0.55)
Forest floor layer				
Control	6.27(0.51)	1.84(0.27)	0.07(0.01)	27.70(0.44)
Low-N	7.43(0.53)	2.26(0.22)	0.08(0.01)	26.75(0.82)
Medium-N	6.63(0.42)	1.85(0.25)	0.07(0.01)	26.32(2.47)
High-N	5.74(1.13)	1.63(0.52)	0.06(0.02)	26.31(0.31)

Table S2 Tropical forest carbon budget in aboveground assessed using different methods (Tg C year^{-1}).

Years	Loss	Gain	Net	References	Notes
2000–2007	2820	2740	-80	Pan et al.2011	(1)
2003–2014	862	437	-425	Baccini et al.2017	(2)
2000-2005	810			Harris et al.2012	(3)
2000-2010	880	97	-783	Achard et al.2014	(4)
2002-2012	1022			Tyukavina et al.2015	(5)
2010-2017	2860	2970	110	Fan et al., 2019	(6)
2000-2007	2800	2700	-100	Mitchard et al., 2018	(7)
2003-2014	1000	500	500	Mitchard et al., 2018	(8)
1990-2007	2900	3000	100	Mitchard et al., 2018	(9)
1980-1990		870		Hubau et al. 2020	(10)
1990-2000		1260		Hubau et al. 2020	(10)
2000-2010		990		Hubau et al. 2020	(10)
2010-2015		730		Hubau et al. 2020	(10)

Notes: (1) Loss: gross deforestation emission, including from soils; Gain: Tropical intact forest+Tropical regrowth forest. (2) Changes in aboveground carbon storage. (3) *Gross; excludes degradation and disturbance. (4) Loss; excludes degradation and disturbance; Gain: Regrowth only; (5) *Loss; excludes degradation and disturbance. (6) Gross: annual aboveground carbon fluxes; Satellite-observed pantropical carbon dynamics. (7) Data are from networks of forest inventory plots6, combined with forest area data from country surveys. (8) Data are obtained from annual 463-m resolution optical satellite data, calibrated using LiDAR data and field plots from the mid-2000s. (9) Data are derived from looking for overlap between atmospheric inversion, modelling and field-plot estimates. (10) Aboveground carbon sink in the intact forests of the pan-tropics.

References:

Y. Pan et al., A large and persistent carbon sink in the world's forests. *Science* **333**(6045), 988-993 (2011).

A. Baccini, W. Walker, L. Carvalho, M. Farina, D. Sulla-Menashe, & R. A. Houghton, Tropical forests are a net carbon source based on aboveground measurements of gain and loss. *Science*, **358**(6360), 230-234 (2017).

N. L. Harris, S. Brown, S. C. Hagen, S. S. Saatchi, S. Petrova, W. Salas, M. C. Hansen, P. V. Potapov, A. Lotsch, Baseline map of carbon emissions from deforestation in tropical regions. *Science* **336**, 1573–1576 (2012).

F. Achard, R. Beuchle, P. Mayaux, et al., Determination of tropical deforestation rates and related carbon losses from 1990 to 2010. *Glob. Change Biol.* **20**, 2540–2554 (2014).

A. Tyukavina, A. Baccini, M. C. Hansen, P. V. Potapov, S. V. Stehman, R. A. Houghton, A. M. Krylov, S. Turubanova, S. J. Goetz, Aboveground carbon l*oss in n*atural and managed tropical forests from 2000 to 2012. Environ. Res. Lett. 10, 074002 (2015).

L. Fan, J.P. Wigneron, P. Ciais, et al., Satellite-observed pantropical carbon dynamics. *Nature Plants* **5**, 944-951 (2019).

E. T. A Mitchard, The tropical forest carbon cycle and climate change. *Nature* **559**, 527–534 (2018).

W. Hubau, S.L. Lewis, O.L. Phillips, *et al.* Asynchronous carbon sink saturation in African and Amazonian tropical forests. *Nature* **579,** 80–87 (2020).

M.G. Kramer, O.A. Chadwick, Climate-driven thresholds in reactive mineral retention of soil carbon at the global scale. *Nature Clim Change* **8,** 1104–1108 (2018).

Table S3 Papers used for meta-analysis on the effects of experimental N additions on soil C and N contents, and soil pH in forest ecosystems in the tropics.

(1) D. Tian, P. Li, W. Fang, et al., Growth responses of trees and understory plants to nitrogen fertilization in a subtropical forest in China. *Biogeosciences 14*(14), 3461-3469 (2017).
(2) Y. Peng, et al., Soil biochemical responses to nitrogen addition in a secondary evergreen broad-leaved forest ecosystem. *Scientific reports* 7(1), 2783 (2017).
(3) Q. Wang, W. Zhang, T. Sun, L. Chen, X. Pang, Y. Wang, F. Xiao, N and P fertilization reduced soil autotrophic and heterotrophic respiration in a young Cunninghamia lanceolata forest. *Agricultural and Forest Meteorology* 232, 66-73 (2017).
(4) X. L. Zhong, et al., Physical protection by soil aggregates stabilizes soil organic carbon under simulated N deposition in a subtropical forest of China. *Geoderma* 285, 323-332 (2017).
(5) W. Zhang et al., High nitrogen deposition decreases the contribution of fungal residues to soil carbon pools in a tropical forest ecosystem. *Soil Biology & Biochemistry* 97, 211-214 (2016).
(6) B. L. Turner, J. B. Yavitt, K.E. Harms, M. N. Garcia, S. J. Wright, Seasonal changes in soil organic matter after a decade of nutrient addition in a lowland tropical forest. *Biogeochemistry* 123(1-2), 221-235 (2015).
(7) Q. Gao, N.J. Hasselquist, S. Palmroth, Z. Zheng, W. You, Short-term response of soil respiration to nitrogen fertilization in a subtropical evergreen forest. *Soil Biology and Biochemistry* **76,** 297-300 (2014).
(8) J. Wu, W. Liu, H. Fan, G. Huang, S. Wan, Y. Yuan, C. Ji, Asynchronous responses of soil microbial community and understory plant community to simulated nitrogen deposition in a subtropical forest. *Ecology and evolution* ***3***(11), 3895-3905 (2013).
(9) X. Lu, F. S. Gilliam, G. Yu, L. Li, Q. Mao, H. Chen, J. Mo, Long-term nitrogen addition decreases carbon leaching in a nitrogen-rich forest ecosystem. *Biogeosciences* 10, 1451-1481(2013).
(10) X. Chen, Y. Li, J. Mo, et al., Effects of nitrogen deposition on soil organic carbon fractions in the subtropical forest ecosystems of s china. *Journal of Plant Nutrition & Soil Science* 175(6), 947-953 (2012).
(11) W. Zhang, X. Zhu, L. Liu, et al., Large difference of inhibitive effect of nitrogen deposition on soil methane oxidation between plantations with N - fixing tree species and non - N - fixing tree species. *Journal of Geophysical Research Biogeosciences* 117(G4), 24-24 (2015).
(12) X. Lu, et al., Nitrogen addition shapes soil phosphorus availability in two reforested tropical forests in southern China. *Biotropica* 44(3), 302-311 (2012).
(13) S. K. Tripathi, C. P. Kushwaha, K. P. Singh, Tropical forest and savanna ecosystems show differential impact of n and p additions on soil organic matter and aggregate structure. *Global Change Biology* 14 (11), 2572–2581(2008).
(14) Y. Huang, R. Kang, J. Mulder, T. Zhang, L. Duan, Nitrogen saturation, soil acidification, and ecological effects in a subtropical pine forest on acid soil in southwest China. *Journal of Geophysical Research: Biogeosciences* **120**(11), 2457-2472 (2015).
(15) B. Koehler, M. D. Corre, E. Veldkamp, H. Wullaert, S. J. Wright, Immediate and long - term nitrogen oxide emissions from tropical forest soils exposed to elevated nitrogen input. *Global Change Biology* **15**(8), 2049-2066 (2009).
(16) M.S. Torn, P.M. Vitousek, S. E. Trumbore, The influence of nutrient availability on soil organic matter turnover estimated by incubations and radiocarbon modeling. *Ecosystems* 8(4), 352-372 (2005).
(17) C. Chen, Z. Xu, J. Hughes, Effects of nitrogen fertilization on soil nitrogen pools and microbial properties in a hoop pine (*Araucaria cunninghamii*) plantation in southeast Queensland, Australia. *Biology and fertility of soils* 36(4), 276-283 (2002).
(18) L. Jiang, et al., The response of tree growth to nitrogen and phosphorus additions in a tropical montane rainforest. *Science of the Total Environment* 618, 1064-1070 (2018).
(19) D. F. Cusack, W. L. Silver, M. S. Torn, W. H. McDowell, Effects of nitrogen additions on above- and belowground carbon dynamics in two tropical forests. *Biogeochemistry* 104(1-3), 203-225 (2011).
(20) J. Cui, J. Wang, J. Xu, C. Xu, X. Xu, Changes in soil bacterial communities in an evergreen broad-leaved forest in east China following 4 years of nitrogen addition. *Journal of Soils and Sediments* 17(8), 2156-2164 (2017).
